# Mesenchymal Stem Cells in Pediatric Physeal Growth Arrest: A Systematic Review

**DOI:** 10.7759/cureus.93064

**Published:** 2025-09-23

**Authors:** Catalina Aloman, Dillon Stone, Rowan Sankar, Ahmed Thabet-Hagag

**Affiliations:** 1 Department of Orthopaedic Surgery and Rehabilitation, Texas Tech University Health Sciences Center - El Paso, Paul L. Foster School of Medicine, El Paso, USA

**Keywords:** bony bar, growth arrest, growth plate, orthopaedics, physeal, physis, stem cells

## Abstract

The physeal growth plate is a common site of involvement in pediatric and adolescent fractures, with 18-30% of fractures complicated by physis injury potentially leading to growth plate arrest and formation of a bony bar (BB). Inadequate treatment of growth arrest in children can cause long-term complications such as limb-length discrepancies or angular deformities. Stem cell transplantation has been increasingly studied as a potential treatment modality for growth arrest and prevention of BB formation in physeal injuries.

The purpose of this study is to summarize the current state of literature on the ability of stem cells to prevent physeal growth arrest, determine the ideal stem cell type for use in physeal injuries, and analyze the methods of stem cell delivery and biochemical environment necessary for stem cell therapy success.

This study is a systematic review. The Preferred Reporting Items for Systematic Reviews and Meta-Analysis (PRISMA) guidelines were utilized to search PubMed including the terms “growth plate arrest”, “physeal injury”, “physeal”, “growth plate injury”, “bony bar”, “stem cell”, “mesenchymal”, and “chondrocyte” from 2012-2024 and then independently analyzed by three reviewers. Studies were excluded if they were off topic, bench-top studies, editorials, non-English, or did not include a focus on stem cells.

The search identified 269 potential articles, 68 remained after applying exclusion criteria, and eight were included after reviewer analysis. No studies in human models remained. Based on the findings of this systematic review, the use of mesenchymal stem cells and chondrocytes, when compared to controls, is associated with a decrease in bony deformities following injury to an open physeal growth plate in animal models. However, there is considerable variability in the literature on the method of stem cell transplantation and which combination of cells provides the greatest benefit.

Given the high likelihood of limb deformity following pediatric or adolescent growth arrest injuries, utilizing mesenchymal stem cells to prevent growth arrest and BB formation would significantly impact the field of pediatric orthopedics. Stem cells have demonstrated the potential to improve outcomes in physeal plate injury in animal models; however, further research is needed to determine the ideal methods and biochemical environment required for successful stem cell therapy in humans.

## Introduction and background

The human physeal growth plate consists of three main layers: reserve zone, proliferative zone, and hypertrophic zone [1]. The reserve zone contains small, scattered mesenchymal stem cells (MSCs) that develop into proliferative chondrocytes (CHCs), the cells primarily responsible for developing cartilage [2]. CHCs then undergo active cell replication in response to growth hormone and also synthesize collagen in the proliferative zone [3,4]. CHCs stop replicating and begin to hypertrophy to later initiate the ossification process controlled by osteoblasts and osteoclasts in the hypertrophic zone [5]. As CHCs proliferate, organize, and hypertrophy, skeletal bones elongate and grow. When this process is halted prematurely prior to reaching bone maturity, typically secondary to an injury or fracture, it is termed growth arrest [6].

Growth arrest can be due to various etiologies such as trauma, infection, neoplasm, congenital disease, or iatrogenic injury involving the reserve layer of MSCs and immature CHCs, which disrupts the normal bone elongation process [7,8]. This causes a physeal bony bar (BB) to form across the growth plate at the point of injury, with fractures involving at least 8% of the growth plate at increased risk [9,10]. If the BB is formed centrally, it will cause the limb to stop growing longitudinally, and if the BB is formed peripherally, it can lead to an angular deformity, with both potentially resulting in limb-length discrepancies [6]. Pediatric fractures involving the growth plate may go unnoticed and require a high degree of clinical suspicion in order to prevent future complications, with the most common injuries occurring in the distal radius and distal tibia [11-13]. Currently, there are multiple accepted methods to correct physeal plate injuries, and the choice between them depends on several factors, including the involved area, age of the child, and BB size and location. However, repeat surgery is required in 60% of cases [6,14,15].

Due to the lack of any current surgical interventions for growth plate arrest injuries that can regenerate damaged physeal tissue, novel interventions that may prevent BB formation and subsequent limb deformity are of great importance in pediatric and adolescent orthopedics. One of the earliest animal models to investigate stem cell transplantation found that sheep CHCs implanted into tibial and femoral growth plate defects could prevent physical BB formation, with subsequent studies demonstrating that scaffolding techniques can improve longitudinal growth [16,17]. Further studies have improved upon these findings; however, their methods include significant variability in the types of cells used, methods to implant them, and biochemical markers. Given the growing body of literature involving stem cell treatment for growth arrest since Shukrimi et al. completed their literature review in 2012, this study aims to (1) analyze ability of stem cells to prevent physeal growth arrest, (2) determine the ideal stem cell type for use in physeal injuries, and (3) analyze the methods of stem cell delivery and biochemical environment necessary for stem cell therapy success [18]. Our question is, are stem cells a feasible treatment option for physeal plate injuries? And our hypothesis states that biochemical and animal models demonstrate initial feasibility; however, further research is needed before implementation into human trials.

## Review

Materials and methods

This systematic review was performed according to the Preferred Reporting Items for Systematic Reviews and Meta-Analysis (PRISMA) recommendations [19].

Literature Search

**Figure 1:**
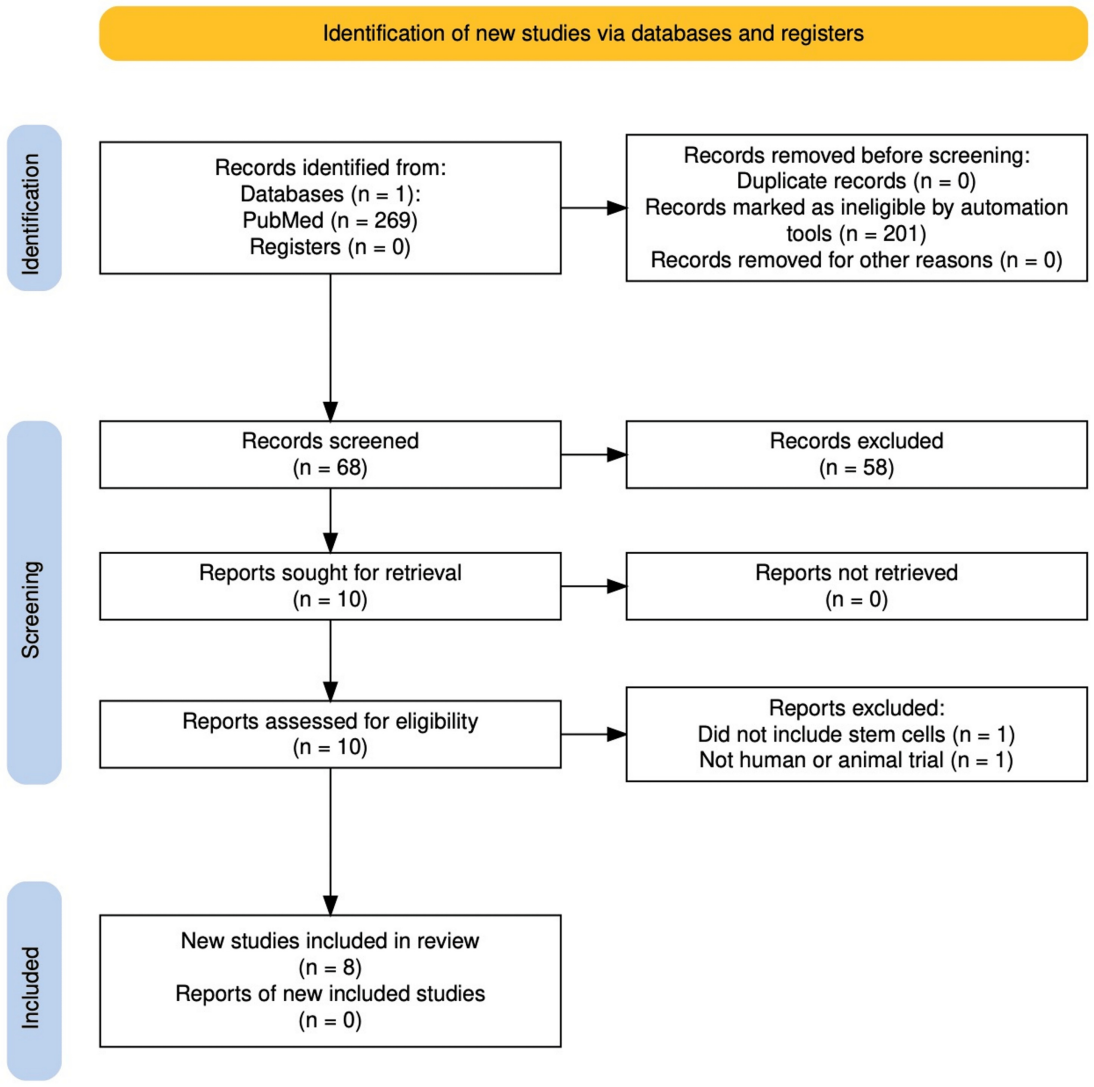
Preferred Reporting Items for Systematic Reviews and Meta-Analysis (PRISMA) flow

A systematic review of the literature was conducted on 1) animal, 2) biochemical, and 3) human studies on growth arrest being treated with stem cells. A PubMed search was conducted, and a PRISMA flow was constructed to show how sources were found (Figure 1) [20].

Inclusion Criteria

A Medical Subject Headings (MeSH) term Boolean search was conducted based on common phrasing for growth plate arrest, including “growth plate arrest”, “physeal injury”, “physeal”, “growth plate injury”, “bony bar”, “stem cell”, “mesenchymal”, and “chondrocyte”, resulting in 269 studies. Filters were applied, including English, full text, human and other animals, and studies from 2012 to 2024, which resulted in 68 articles remaining.

Exclusion Criteria

Studies were first excluded if they were off topic (for example, studies on cell cycle arrest or yeast growth arrest), editorials, or reviews (focusing on recent trials only). During the full text evaluation phase, studies were excluded if they did not pertain to stem cell treatment of growth arrest with a focus on animal or human studies, including radiological or histological results. In an effort to include all pertinent material, the MeSH search terms were left broad, and exclusion was completed by the three reviewers (RS, CA, FM) by ascertaining the relevance of the paper examining both growth arrest of physeal plates and the use of stem cells.

Search Strategy and Study Selection

The PubMed database was used as the primary search source, which has MEDLINE and Cochrane articles indexed within it. The search results were exported to Microsoft Excel (Microsoft® Corp., Redmond, WA) for cataloguing and removing duplicates. Once the duplicates were removed, the remaining abstracts and full articles were read using the aforementioned inclusion criteria by three reviewers (RS, CA, FM).

Data Extraction and Quality Appraisal of Included Studies

Three reviewers (RS, CA, FM) were responsible for qualitatively analyzing each article for the specific study aims. The data from each study were extracted and recorded in Microsoft Excel to evaluate for patterns and relationships in the data. Each of the studies was evaluated for quality by the three reviewers as well.

Results

Study Selection

A total of 269 articles related to growth arrest and stem cells were reviewed utilizing the PRISMA guidelines (Figure 1). Of these, 68 remained after applying the inclusion and exclusion criteria. The abstracts of all 68 articles were evaluated on the first pass, looking for animal or clinical trials and the use of stem cells, after which 10 full-text articles were reviewed. One article was excluded for not utilizing stem cells in their study design, and one article was excluded for not being an animal or human trial, leaving eight studies remaining for qualitative analysis. The eight studies were published between 2012 and 2022 and were performed in the United States of America, Italy, Turkey, Iran, China, Japan, Singapore, and the Czech Republic. None of the included studies involved humans.

Risk of Bias

Risk of bias was assessed with the Systematic Review Centre for Laboratory Animal Experimentation (SYRCLE) tool [21]. This framework is specific to animal experimentation and is subjectively rated by the reviewer on a high/low/unclear scale [21]. The risk of bias was assessed by two reviewers (CA, DS). The original article recommends against assigning summary ratings to each article. Full details regarding the risk of bias assessment can be found in Table 1 [21].

**Table 1:** Systematic Review Centre for Laboratory Animal Experimentation (SYRCLE) risk of bias tool for animal studies

Author	Sequence Generation	Baseline Characteristics	Allocation Concealment	Random Housing	Blinding (Caregivers/Researchers)	Random Outcome Assessment	Blinding (Outcome Assessor)	Incomplete Outcome Data	Selective Outcome Reporting	Other Sources of Bias
Planka et al., 2012 [17]	High	Low	High	Unclear	High	Low	Unclear	Low	Low	Low
Yoshida et al., 2012 [22]	Unclear	Unclear	High	Unclear	High	Low	Unclear	High	Low	Low
Coleman et al., 2013 [23]	High	Low	High	Unclear	High	Low	Unclear	Low	Low	Low
Azarpira et al., 2015 [24]	High	Low	High	Unclear	High	Low	Unclear	Low	Low	High
Clark et al., 2015 [25]	Unclear	High	High	Unclear	High	Low	Unclear	Low	Low	Low
Lee et al., 2016 [26]	High	Low	High	Unclear	High	Low	Unclear	Low	Low	Low
Gültekin et al., 2020 [27]	Unclear	Low	High	Unclear	High	Low	Unclear	Low	Low	Low
Wong et al., 2022 [28]	Unclear	Low	High	Unclear	High	Low	Unclear	Low	Low	Low

Results of the Biochemical Studies

In addition to the stem cells and delivery methods this review focused on, the cellular signaling molecules and pathways that play a role in growth arrest were also evaluated. Musumeci et al. found that the inflammatory cytokines interleukin-6 and tumor necrosis factor alpha (TNF-α) play large roles in recruiting neo-formed bone trabeculae to the growth plate following an insult that leads to growth arrest [29]. Additionally, caspase-3 and PARP-1 proteins were associated with higher rates of CHC apoptosis at the physeal plate after injury, contributing to growth arrest [29]. Similarly, Karuppaiah et al. reported that increased Fibroblast growth factors 9 and 18 led to increased fibroblast growth factor receptor 3 signaling in growth plate CHCs that suppressed CHC proliferation [30]. Finally, damaged DNA binding protein 1 (DDB1) knockout mice were found by Zhao et al. to induce p27 upregulation at the growth plate and subsequent cell cycle arrest in primary CHCs [31].

Results of the Animal Studies

All eight of the reviewed studies demonstrated the use of stem cells to treat iatrogenic traumatic growth plate injuries. The most common method of inducing the injury was with a high-speed drill burr to control the size and shape of the defect as described by Yoshida et al. [22]. The defects ranged in size from 1 mm to 12 mm [17,25]. Azarpira et al. removed the largest amount of the growth plate: 50% of the lateral distal femoral physis [24]. 

Within this systematic review, MSCs were the most commonly used cells being utilized in seven of the eight papers in some way (Table 2) [17,22-25,27,28]. Lee et al. were the only group that did not, opting to utilize only CHCs in their trial with similar results to the others [26]. Planka et al., Yoshida et al., and Gültekin et al. used MSCs in combination with CHCs or other extracellular matrix tissue [17,22,27]. Seven of the eight studies utilized a single control group that replicated the method of stem cell delivery without the stem cells [17,22-24,26,27]. However, Clark et al. had a separate control group with a scaffold seeded with insulin-like growth factor-1 (IGF-1) [25]. They found that the rabbits implanted with the bone marrow stem cells had more growth than the IGF-1 control [25].

**Table 2:** Summary of results from animal studies using stem cells to treat growth arrest BMC: autologous bone marrow cell; BMSC: bone marrow-derived stromal cell; CHC: chondrocyte; MSC: mesenchymal stem cell; PBS: phosphate-buffered saline solution; TEC: tissue engineering construct

Authors	Type of Study	Source of Stem Cells	Types of Stem Cells	Delivery Method	Region of Defect	Deformity Measurements	Deformity Results	Histological Results
Planka et al., 2012 [17]	Animal	Pig os ilium allogeneic bone marrow	MSCs and chondrocytes (CHCs)	Collagen-chitosan scaffold with allogeneic MSCs and CHCs (three blocks)	Distal femur	Length of bone. angular deformity of distal femur	Longer femoral length and less valgus angular deformity with MSCs and CHC scaffold	Bony bridge formation prevented and newly formed type 2 collagen with signs of columnar organization detected
Yoshida et al., 2012 [22]	Animal	Rabbit knee joint synovial tissue	MSCs and extracellular matrix (ECM)	Scaffold-free tissue-engineering construct from MSCs	Proximal tibia	Medial proximal tibial angle	Lesser angular deformity noted in experimental groups	Greater regeneration of the growth plate with MSCs in the TEC differentiating into proliferative and prehypertrophic CHC-like cells
Coleman et al., 2013 [23]	Animal	Rat femoral and tibial bone marrow	Bone marrow-derived stromal cells (BMSCs)	BMSCs were suspended in an agarose solution	Distal femur	Femoral length, medial proximal tibial angle, lateral proximal tibial angle	Limb length discrepancy between injured and controlled limbs was corrected with undifferentiated MSCs	Decreased mineralized tether formation in the growth plate tissue around the defect to normal levels
Azarpira et al., 2015 [24]	Animal	Rabbit iliac wing bone marrow	Mesenchymal stem cells (MSCs)	MSCs within a chitosan scaffold	Distal femur/patella	Distal metaphyseal angle	Less angular deformity with MSCs in chitosan scaffold	More tissue maturity and better growth response with MSCs
Clark et al., 2015 [25]	Animal	Rabbit tibial diaphyseal bone marrow	Autologous bone marrow cells (BMCs)	Porous poly(lactic-co-glycolic acid) scaffold with BMCs was into resected bony bar	Proximal tibia	Medial proximal tibial angle, lateral proximal tibial angle	No significant reduction in the angular deformity was noted, but there was decreased bony bar formation	Increased CHC population and a reduced loss of the remaining native growth plate
Lee et al., 2016 [26]	Animal	Rabbit femoral head and tibial plateau bone marrow	CHCs	CHCs within a scaffold-free cartilage tissue free analogue	Proximal tibia	Medial proximal tibial angle	Subtle tibial varus deformity with CHCs compared to significant tibial varus deformity with scaffold alone	Less bone bridge formation with regenerated tissue in the columnar arrangement of normal physeal tissue
Gültekin et al., 2020 [27]	Animal	Rabbit femoral condyle and iliac crest bone marrow	MSCs and CHCs	Sheets of MSCs were placed onto the injury site	Proximal tibia	Tibial length, medial proximal tibial angle	Significant intergroup difference of limb length and medial proximal tibial angle	Bony bridge and pathologic fibrosis formation over the growth plate were prevented
Wong et al., 2022 [28]	Animal	Immortalized E1-MYC 16.3 human embryonic human stem cell-derived MSC line	MSCs	A single intra-articular injection of 100 µg of MSC exosomes in 100 µL of phosphate-buffered saline solution (PBS)	Distal femur	Femur limb length	Significantly decreased limb length discrepancy between MSC + PBS vs. just PBS	Significantly more CHCs, sulfated glycosaminoglycan, and collagen II than PBS-treated defects. Bone bridge formation present in both groups.

The use of these MSCs in isolation and combination with CHCs led to practically and statistically significant improvements in limb and histologic measurements of growth plate injuries. Six of the eight animal trials found significantly decreased medial proximal tibial angle deformity, significantly decreased distal metaphyseal angle deformity, and significantly less varus deformities of the leg when compared to the controls [17,22-24,26,27]. Additionally, Planka et al., Coleman et al., and Gültekin et al. all found the leg treated with implanted stem cells to be significantly longer in length than the controls [17,23,27]. Clark et al. stated that although there was a decreased BB formation across the growth plate, there was no statistically significant difference in angular deformity noted [25]. Wong et al. had no difference in BB formation but did note increased CHCs, sulphated glycosaminoglycan, and collagen II in the MSC exosome-treated group than PBS-treated (phosphate-buffered saline solution) defects [28]. Of note, this study was the only one to use MSC exosomes and not full stem cells [28].

The method of stem cell delivery was much more variable than the types of stem cells used (Table 2). Planka et al., Azarpira et al., and Clark et al. utilized a chitosan or poly (lactic-co-glycolic acid) scaffold to implant the MSCs into the growth plate defects [17,24,25]. Gültekin et al. opted for placing sheets of MSCs and CHCs over the physeal plate defects [27]. Coleman et al. suspended the bone marrow-derived stem cells in an agarose solution, which was inserted into the defect [23]. Wong et al. utilized intra-articular injections of the MSC exosomes in a phosphate-buffered saline solution [28]. Finally, Yoshida et al. and Lee et al. designed a scaffold-free tissue-engineered construct that held the MSCs and CHCs within the physeal plate defects [22,26]. 

Discussion

Recent research into open physeal plate injuries details implanting stem cells into the physeal plate after the insult has occurred, which has been shown to potentially recreate natural growth. Shukrimi et al. reviewed the literature in 2012 and found several animal studies showing the positive effects of MSCs on treating growth arrest injuries in pigs, rabbits, and sheep [18]. This review aims to analyze the growing body of research conducted since 2012.

The injuries studied in all of the articles found in this review utilized iatrogenic trauma to the distal femoral and proximal tibial growth plates. Although trauma is the most common cause of growth arrest in children and adolescents, the growth arrest process is fairly uniform despite the etiology [6]. This review demonstrates that the use of MSCs in growth arrest injuries enables earlier intervention for much more severe cases of growth arrest. Currently, it is nearly impossible to completely prevent BB formation and subsequent limb deformity if more than 10-20% of the growth plate is involved [9]. Foster et al. were only able to achieve regrowth using sheets of CHCs in defects involving approximately 20% of the growth plate [16]. However, Azarpira et al. have shown that using stem cells could prevent BB formation and preserve growth plate function in injuries where up to 50% of the growth plate is impacted [24]. Furthermore, depending on the type of cell and method of delivery, it may be possible to recover CHC organization and hypertrophy in the growth plate in even larger defects. Planka et al. achieved cartilage regrowth and deformity decrease with defects as large as 12 mm [17]. Although they did not estimate the percentage of the growth plate of their defect takes up, depending on the size of the rabbits used, it may be as high as 60% [32]. This treatment option could present new opportunities for fractures that are currently difficult to treat, including highly comminuted distal femur and proximal tibia fractures; however, further research is still needed to detail exactly which fractures and fracture patterns could be amenable to stem cell implantation. These results signal promising initial evidence that MSC implantation is a viable method to prevent BB and subsequent deformity formation in high-involvement growth arrest injuries.

Types of Stem Cells

The type of stem cells used in treating growth arrest injuries has been fairly consistent. Lee et al. were the only group who chose to use differentiated CHCs cultured from MSCs instead of undifferentiated stem cells [19]. Although they reported statistically significant differences in deformity correction and BB prevention, more trials directly comparing differentiated to undifferentiated stem cell implantation are necessary. Based on the results of this review with multiple successful outcomes in animal models, it appears that stem cell use in physeal growth plate injuries shows promise for future implementation into human trials. Additionally, more research is necessary to demonstrate the efficacy of undifferentiated MSCs implanted with differentiated CHCs or other extracellular matrix tissue. Yoshida et al. hypothesize that the additional extracellular matrix tissue more precisely replicates the biochemical environment of the undamaged growth plate [22]. Although all eight studies found decreased growth plate disruption at the cellular level, it is not known if culturing MSCs with other cell lines would have a summative effect on outcomes. Both have been shown to be successful; however, for this technique to be testable in humans, more research needs to be conducted directly identifying what combination of cells leads to the greatest deformity prevention.

Implanting Stem Cells

The methods of implanting the stem cells into growth arrest defects were significantly more variable than the types of cells used. Despite seven of the eight studies utilizing the same cell line, there were four different methods of implanting the cells, with only three of the eight studies implementing a similar scaffold method [17,22,25]. Additionally, each of these scaffolds was completely different in design. Planka et al. described using a chitosan scaffold with a three-block structure that housed one group of MSCs in the middle with two groups of CHCs on either side [17]. Clark et al. developed a unique scaffold structure using porous poly (lactic-co-glycolic acid) that was not seen anywhere else in the literature [25]. While the scaffold was a popular option, Yoshida et al. demonstrated the use of a novel engineered tissue construct that does not rely on a supporting scaffold for the implantation of stem cells into the growth plate [22]. Lee et al. also developed their own cartilage tissue analog [26]. Despite the various novel methods to deliver stem cells into the growth plate, there were no comparisons between the types of scaffolds or between any of the different implantation methods. This lack of consensus remains a large hurdle for translating this technique into human trials.

Current Treatment Options

While research on stem cell use in pediatric physeal plate injuries remains in the preclinical phase, a discussion of current treatment options is included. One of the most common methods of physeal injury repair includes BB resection followed by implantation of fat, muscle, cement, or bone to prevent BB reformation [33]. This technique has successfully treated physeal injuries in younger children (with more than two years of bone growth remaining) and with a smaller BB size. However, this method is associated with poor outcomes in older children and patients with increased BB size [34]. For growth plate injuries that are not viable surgical candidates for BB resection, more invasive options can be utilized, which are associated with similarly successful outcomes but with longer recovery times [35].

Chrondrodiastasis involves breaking the bone at the place of least resistance and then using progressive fixation to induce bone growth at the epiphysis. This therapy is painful for patients and requires numerous invasive procedures involving months to years of external fixation [34]. Additionally, the results of chrondrodiastasis are highly variable, so this technique is usually reserved for older children with less growth potential remaining [36]. Finally, if the growth plate injury was not caught before the fusion of the diaphysis to the epiphysis, the only option for intervention is deformity correction. These procedures correct angular deformities or limb-length discrepancies after they have formed. However, these procedures are invasive and are often unsuccessful in full deformity correction [37]. Efforts to improve outcomes were described by Manoudis et al., showing that concurrent autologous iliac crest tri-cortical bone grafting, along with late fibular length deformity correction, sped up the recovery process [38].

Biochemical Environment

Although this review mainly focused on the use of stem cells to treat growth arrest, there were three articles included that reported on the biochemical setting of growth arrest and the cellular signals that play a role in inducing growth arrest. While not specific to stem cells, this information was felt to be valuable enough to include in a review about treating growth arrest. Understanding the regulators of the physeal plate environment is critical information for future researchers investigating the effect of stem cells on growth arrest. Musumeci et al.’s findings of increased interleukin-6 and TNF-α associated with increased CHC apoptosis may provide clues to augment stem cell therapies [29]. Immunotherapies that target TNF-α may be able to decrease the chance of stem cell implantation failure by creating a microenvironment that suits the formation of CHCs [39]. Additionally, Karuppaian et al.’s discussion of the inverse relationship between fibroblast growth factor receptor 3 expression and CHC proliferation has been demonstrated in vitro, reinforcing the need to downregulate this signaling pathway in growth arrest injuries [30,40]. Although the implantation of stem cells in isolation was shown to prevent BB formation, understanding how to augment this technique will significantly increase the viability and success of interventions involving stem cells. Clark et al. demonstrated that MSCs implanted with IGF-1 achieved the greatest reduction in limb deformity and the most cartilage growth at the physeal plate [25]. Although further investigations are needed, the knowledge about the influence of growth factors on growth arrest may provide a powerful adjunct therapy to MSC implantation.

Clinical Correlations and Future Directions

Given the high prevalence of the debilitating complications of growth arrest injuries, a successful and consistent intervention would change the landscape of pediatric and adolescent orthopedics for the better [41]. These injuries are associated with significant deformity and can require follow-up surgeries, which are invasive and are not without complications [15]. This review demonstrates the potential impact of implanting MSCs into the growth plate at the time of insult. Planka et al. showed that the angular deformity of a growth arrest injury treated with MSCs in a chitosan scaffold was 1.4 degrees compared to the scaffold alone, which was 4.5 degrees on average [17]. Furthermore, they demonstrated that MSCs led to femoral lengths 29% longer than the control after growth arrest [17]. The use of stem cells to treat growth arrest would provide a significantly better quality of life to adolescents who sustained a growth plate injury. Gültekin et al. showed that after bony bridge formation and resection, stem cells can restore the natural growth plate histology with prevention of further fibrosis and BB formation at the growth plate [27]. This would suggest that surgical removal of the BB would still be required in these patients if it has already begun to form with the stem cells, then acting as an adjunct treatment to restore normal physeal histology and promote normal growth thereafter. This would be critical for those children who may have had their growth plate injury initially missed and experienced resultant BB formation. The use of MSCs may provide an alternative to the multiple painful operations often required for deformity correction procedures.

Limitations

This study was limited in scope as there are no current or past human trials. As a result, these conclusions may not be directly applicable to patient care, but it is our hope that these findings aid in spurring further research into this topic. Additionally, many of the included studies report different information, preventing the pooling of results. Standardized reporting regarding histologic, radiographic, and clinical outcomes would greatly enhance the ability to analyze findings.

## Conclusions

Despite the abundance of successful animal studies using stem cells to reduce deformity in growth plate arrest, there is a specific need for experimental trials to develop clinical guidelines for the use of MSCs in children and adolescent growth arrest injuries. It is important to note that despite a comprehensive review of the literature, there were only eight studies in the past 12 years that investigated stem cells as a treatment for growth arrest injuries in animal models and included no human trials. The conclusions derived from these articles, while valuable, also emphasize the lack of knowledge about how best to use stem cells and other biochemical modulators to treat growth arrest injuries in human patients. It reinforces the importance of continued research on stem cells, implantation techniques, the growth plate environment, cost analysis, and specific indications for this intervention. From these results, we hope to provide a solid foundation for spurring further research into human trials with the end goal of creating a new standard of care for pediatric and adolescent physeal plate injuries.
